# The influence of raw milk exposures on Rift Valley fever virus transmission

**DOI:** 10.1371/journal.pntd.0007258

**Published:** 2019-03-20

**Authors:** Elysse N. Grossi-Soyster, Justin Lee, Charles H. King, A. Desiree LaBeaud

**Affiliations:** 1 Department of Pediatrics, Division of Infectious Diseases, Stanford University School of Medicine, Stanford, CA, United States of America; 2 Quantitative Sciences Unit, Stanford University School of Medicine, Stanford, CA, United States of America; 3 Center for Global Health and Diseases, Case Western Reserve University, Cleveland, OH, United States of America; Faculty of Science, Ain Shams University (ASU), EGYPT

## Abstract

Rift Valley fever virus (RVFV) is a zoonotic *phlebovirus* that can be transmitted to humans or livestock by mosquitoes or through direct contact with contaminated bodily fluids and tissues. Exposure to bodily fluids and tissues varies by types of behaviors engaged for occupational tasks, homestead responsibilities, or use in dietary or therapeutic capacities. While previous studies have included milk exposures in their analyses, their primary focus on livestock exposures has been on animal handling, breeding, and slaughter. We analyzed data from multiple field surveys in Kenya with the aim of associating RVFV infection to raw milk exposures from common animal species. Of those with evidence of prior RVFV infection by serology (n = 267), 77.2% engaged in milking livestock compared to 32.0% for 3,956 co-local seronegative individuals (p < 0.001), and 86.5% of seropositive individuals consumed raw milk compared to 33.4% seronegative individuals (p < 0.001). Individuals who milked and also consumed raw milk had greater odds of RVFV exposure than individuals whose only contact to raw milk was through milking. Increased risks were associated with exposure to milk sourced from cows (p < 0.001), sheep (p < 0.001), and goats (p < 0.001), but not camels (p = 0.98 for consuming, p = 0.21 for milking). Our data suggest that exposure to raw milk may contribute to a significant number of cases of RVFV, especially during outbreaks and in endemic areas, and that some animal species may be associated with a higher risk for RVFV exposure. Livestock trade is regulated to limit RVFV spread from endemic areas, yet further interventions designed to fully understand the risk of RVFV exposure from raw milk are imperative.

## Author summary

Part of the transmission cycle for Rift Valley fever virus (RVFV) is related to direct human interaction with animals as part of everyday activities, including consumption of animal products for nutritional or therapeutic benefits. Although the vector-borne transmission of RVFV by mosquito populations is well understood, less is known about how human contact with animal tissues and fluids yields direct (non-vector-borne) RVFV transmission. This study describes the risks of RVFV transmission contributed by exposure to raw milk. It analyzed humans’ milk-related activities and their cumulative risk of RVFV infection, as determined by community-based behavioral and serological surveys in four villages in Kenya. Our data suggest that likelihood of exposure is increased both by actively milking live animals and by direct consumption of raw milk. The risk of RVFV exposure varied among the species of animals kept as livestock and utilized for milk production. Further investigations are necessary to fully characterize the dynamics of RVFV in milk. A better understanding of the role of milk in RVFV transmission will contribute to the public health management of RVFV outbreaks and interepidemic infections.

## Introduction

Rift Valley fever virus (RVFV) is an RNA virus of the *Phenuiviridae* family [1, 2] that causes a wide range of disease symptoms in both humans and animals [3–6]. Originally isolated in 1930 in the Rift Valley of Kenya [7, 8], RVFV remained within the continent of African until 2000 when it emerged in Saudi Arabia and Yemen [8–11]. RVFV is endemic in much of sub-Saharan Africa [3, 12–19] and the Arabian Peninsula [3, 20–22], but imported cases, fueled by travelers from Europe [23, 24] and more recently, China [25, 26], continue to raise concerns of a future major emergence of RVFV to currently unaffected areas. Many studies theorize that the long-term isolation of this virus combined with changing climate patterns and the ubiquitous availability of vector species suggests it has built substantial momentum for emergence in naïve populations and regions [27–31], as seen recently with other mosquito-borne viruses including West Nile virus (WNV) [32, 33], chikungunya virus (CHIKV) [34–36], and Zika virus (ZIKV) [34, 37, 38].

Infection with RVFV can cause high fever (40°C– 42°C), nasal discharge, vomiting, and injected conjunctivae in animals [24, 39, 40]. Similar symptoms of nonspecific febrile illness are common in humans, with risk of severe sequelae, including retinitis and reduction or complete loss of vision, acute hepatitis, renal failure, hemorrhagic disease, encephalitis, and neurological complications [11, 24, 41–44]. While the spectrum of animal species that are susceptible to RVFV infection is quite extensive, sheep are considerably susceptible, followed by other common domesticated livestock species such as cattle and goats [45, 46]. Newborn and younger animals are at a higher risk for severe disease and death, generally within 5 days, as a result of RVFV infection [46–48]. Humans may experience asymptomatic infection, which is rarely diagnosed or reported, and may contribute to the spread of infection. Causative factors influencing variability of disease presentation in humans have yet to be determined.

Fatality rate also increases in naïve animal populations, which can lead to a sudden devastation of herds. Multiple generations of animals can be lost during outbreaks as pregnant animals often experience spontaneous abortion, making post-infection herd recovery difficult. Rapid reduction in herd size can also have significant financial and resource burdens for families and villages that rely on income from the sale of animal meats, milks, and byproducts. Currently, import and export of meat and livestock is governed by the World Organization for Animal Health (OIE) Terrestrial Animal Health Code [49], which restricts trade after detection of clinical signs or laboratory-confirmed case of RVFV. Under these regulations, formal importation of milk and milk products requires presentation of an international veterinary certificate attesting that the products have been pasteurized and hygienic practices and control measures were met for each product imported from countries not free from RVFV [49]. Such guidelines do not apply to individual behaviors or intercountry trade and sales, and therefore do not restrict products that may be contributing to the maintenance of inter-epidemic.

Vector-borne transmission of RVFV is widely understood [11, 50–53], and there are many opportunities for direct zoonotic transmission in populations that engage in regular animal handling, breeding and rearing, and slaughtering. Direct contact with high volumes of animals for slaughter, such as one would find in an abattoir or slaughterhouse, has been shown to increase risk for exposure to RVFV when compared to regular behaviors associated with keeping animals at the homestead [14]. Similarly, handling abortus from infected animals carries significant risk for exposure, as the aborted fetus may contain a high titer of live virus [54].

While the mosquito-borne transmission cycle of RVFV is well understood[55–59], many gaps in the knowledge of other mechanisms of RVFV transmission persist, specifically underlying risk factors that may contribute to interepidemic transmission and emergence in new regions. Regular exposure to potentially infected animals contributes to the incidence of human RVFV infections, yet the parameters of human behaviors and animal exposure have not been well defined. Our study aimed to characterize the importance of raw milk and behaviors related to milk exposures as a previously understudied method of zoonotic RVFV transmission.

## Materials and methods

### Study area

**Fig 1:**
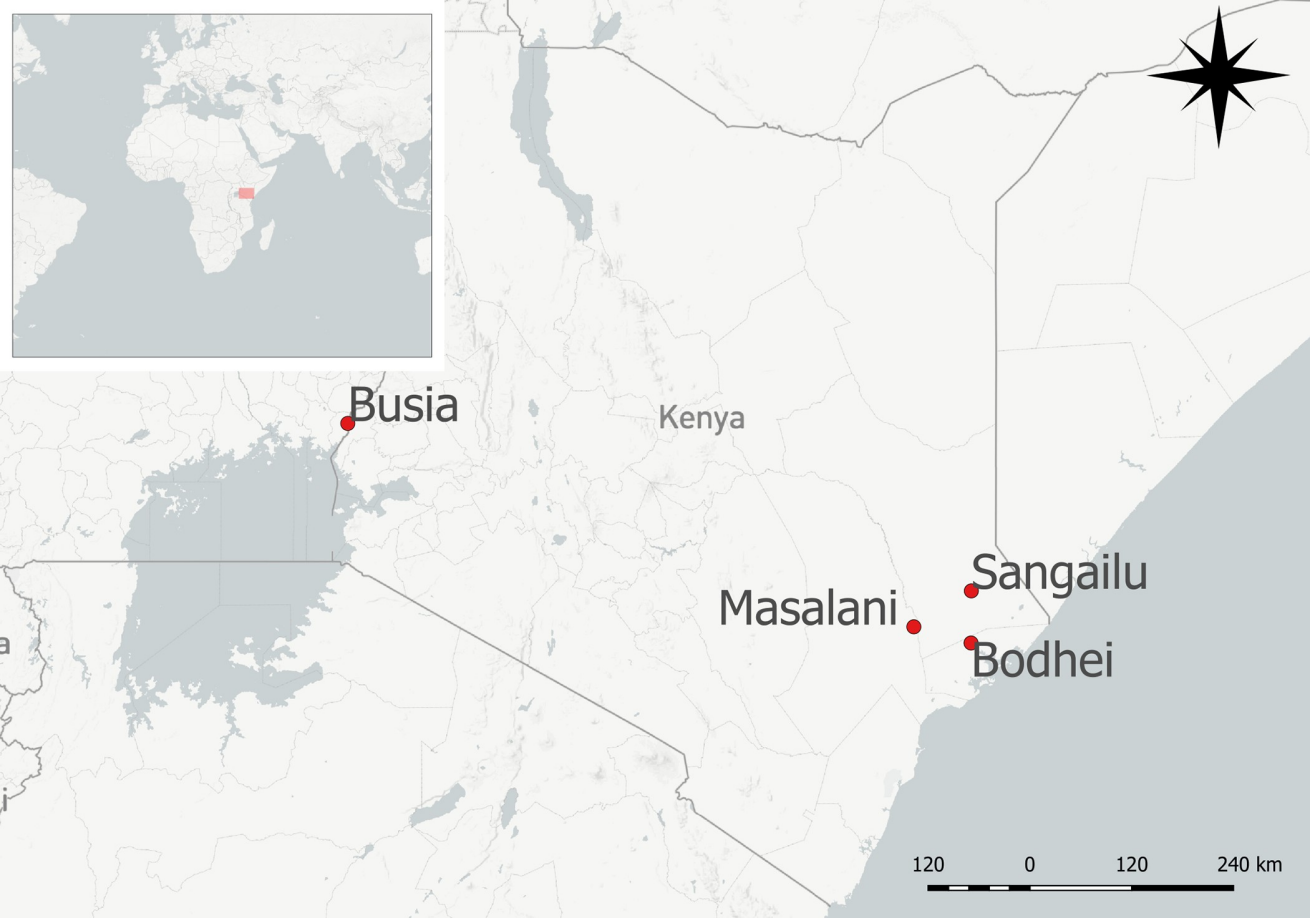
Study area. Kenya is identified in the red square in the upper left map of Africa and surrounding geographical areas. The main map is a close-up map of Kenya that specifies the locations of each region or village wherein participants were enrolled and surveyed to be included in the original studies. Each village region is indicated by a red circle and labeled with the region or village name. Created in QGIS 2.18.11 using MapBox.

This secondary analysis describes populations in two specific regions of Kenya: Western and Eastern Kenya (Fig 1). Each participating village provides a unique perspective to risks associated with RVFV exposure, as village practices are unique to their surrounding environment, bordering regions, and available resources.

Participants in the Western region resided within 45 kilometers of Busia, Kenya, with notable borders of Uganda and Lake Victoria, as previously described [14]. Enrollment and sample collection occurred as a part of a zoonoses study from 2010 to 2012. The Busia region is primarily rural with considerable representation of Luo, Luhya, and Teso ethnic groups in the area [14]. Keeping livestock is common on the homestead or village level, and many participants confirmed regular contact or behaviors with animals.

Eastern region locations of Kenya included in this analysis had three main clusters: Bodhei, Sangailu, and Masalani. The sampling cluster from Garissa county [60], included a collection of smaller villages within the Sangailu region, including Golabele, Sabenale, Gedilun, Matarba, Korahindi, and Tumtish [44]. Villages within Sangailu are predominantly semi-nomadic pastoralists and herders, many of whom are of Somali ethnicity. Enrollment and data collection within Sangailu occurred between August and November of 2011 [44]. Enrollment and data collection in Bodhei, a predominantly forested area of Lamu County, occurred in 2006 during an interepidemic period for RVFV [60]. Masalani is a semi-arid region located in the Ijara constituency of Garissa County [61, 62]. Populations sampled in Masalani included the rural village of Gumarey and the larger town of Sogan-Godud [61, 62]. Participants enrolled in Masalani were sampled in two phases: during an interepidemic period in early 2006 [61], and a post-epidemic period in late 2009 [62]. Sampling was not conducted during active outbreaks in any of the villages included in this secondary analysis, as the only reported outbreak in Kenya occurred in late 2006 to early 2007 [58, 63, 64].

### Ethical approval

Ethical approval was obtained for the primary community surveillance studies individually. Studies conducted in Bodhei and Masalani, titled “Late Outcomes of Rift Valley Fever in Kenya: Ijara Clinical Survey” were reviewed and approved by the University Hospitals Institutional Review Board (IRB) for Human Investigation at Case Western Reserve University (Protocol #10-04-09) and the Kenya Medical Research Institute (KEMRI) Ethical Review Committee (SSC Protocol #918). Ethical approval for the study conducted in Sangailu, titled “Innate Immune Factors in Host Susceptibility to Rift Valley Fever Virus” was granted from the University Hospitals Case Medical Center IRB for Human Investigation at Case Western Reserve University (UH IRB #: 11-09-01) and the KEMRI Ethical Review Committee (SSC Protocol #195). Ethical approval for the “People, Animals and their Zoonoses (PAZ) project community and slaughterhouse worker studies conducted in Busia were obtained by the KEMRI Ethical Review Committee (SSC Protocols #1701 and #2086). Written, informed consent was obtained from all participants for each primary study using consent forms that were available in English and Kiswahili. For child and adolescent participants (ages 1–17), a parent or legal guardian provided consent. All approved consent forms presented a section on “future use” of samples and data collected, and all of the participants included in this secondary analysis approved continued usage for future studies.

### Surveys and serological analysis

This study was a pre-specified secondary analysis applied to multiple existing datasets. Participants aged 1 to 87 years were enrolled in previous cross-sectional studies to determine the prevalence of past RVFV exposure using standard serological methods. All participants were administered a questionnaire uniquely designed for the primary goals of each study to detail basic demographic data, health history, and epidemiologic data regarding lifestyle, environmental exposures, and other behaviors.

Serological status was determined by indirect IgG enzyme-linked immunosorbent assay (ELISA) at the time of the original studies, as previously described [14, 15, 44, 61, 62]. All serology was performed according to the same ELISA protocol, using control serum verified by plaque reduction neutralization testing (PRNT).

### Statistical analysis

This secondary study specifically aimed to investigate the statistical importance of raw milk-related exposures for risk of RVFV exposure and transmission. All primary studies were specific to investigating RVFV in Kenya, and utilized questionnaires regarding daily activities and behaviors, occupational behaviors, and specific questions relating to animal exposures and the consumption of animal products, such as meats and milks. For this secondary analysis, data specific to behaviors relating to milk exposure and serology for RVFV from each separate survey study were compiled using Excel. Statistical analyses of the exposures, specifically milking versus consuming or ingesting raw milk from specific animal species were performed using R version 3.3.1 [65].

Exposure methods (milking and consumption) were modeled separately by logistic regression while only adjusting for age and gender. Forest plots were created to illustrate age and gender adjusted odds ratios (OR) of RVFV infection for each exposure with a 95% confidence interval (CI_95_). Additionally, full models were fit to: (1) examine the potential impact of geographic bias and (2) include an interaction term for milking and consumption to tease out the impact of exposure to one or both exposure methods for Tables 2 and 3, respectively.

Standardized Mean Difference (SMD) was calculated using the “tableone” package in R [65]. SMD was used to visualize the distance between two groups by standardizing variables, specifically those with prior RVFV exposures, or “infection”, and those without prior RVFV exposure, or “no infection”, standardized by animal type.

## Results

Demographic data and behavioral data relating to milk were analyzed for potential risk factors for RVFV exposure, as reported in Table 1. Prior exposure to RVFV varied among villages included in this study. Of the four main regions included in this study, prevalence of RVFV infections ranged from 62.9% (Sangailu (n = 168)) to 10.9% (Busia, n = 29), indicating variability across the villages within our study site (p < 0.001, SMD = 1.37). The age of participants included in the total cohort skewed towards young adult (mean = 26.80 years of age, standard deviation (SD) = 19.64), yet the mean age of individuals with history of RVFV infection was 40.70 (SD = 19.41), which is comparatively higher than the mean age of those without prior infection (mean age = 25.04, SD = 19.24) (p < 0.001, SMD = 0.811). The youngest participant to test seropositive for prior RVFV infection was 2 years old, and the oldest was 85 years old. Gender was not found to be a considerable factor in RVFV exposure history, as approximately half of each exposure cohort identified as female (p = 0.072, SMD = 0.118).

**Table 1:** Demographic and milk exposure factors associated with RVFV exposure.

		Prior Exposure to RVFV			
Characteristics	Total Cohort (n = 4,223)	No Infection (n = 3,956)	Infection (n = 267)	*p* value	SMD
Village–n (%)				<0.001	1.373
Bodhei	206 (4.9)	170 (4.3)	36 (13.5)		
Busia	2,634 (62.4)	2,605 (65.8)	29 (10.9)		
Masalani	249 (5.9)	215 (5.4)	34 (12.7)		
Sangailu	1,134 (26.9)	966 (24.4)	168 (62.9)		
Region–n (%)				<0.001	1.371
West	2643 (62.4)	2605 (65.8)	29 (10.9)		
East	1580 (47.6)	1351 (34.2)	238 (89.1)		
Age–(mean (SD))	26.03 (19.62)	25.04 (19.24)	40.70 (19.41)	<0.001	0.811
–(median (IQR))	21.0 (9.0, 39.0)	19.0 (8.0, 87.0)	40.0 (26.5, 52.5)		
Female–n (%)	2,012 (47.6)	1,870 (47.3)	142 (53.2)	0.072	0.118
Milking–n (%)*					
Any	1,470 (34.8)	1,264 (32.0)	206 (77.2)	<0.001	1.024
Cow	1,219 (28.9)	1,026 (25.9)	193 (72.3)	<0.001	1.052
Sheep or Goat**	1,000 (23.7)	799 (20.2)	201 (75.3)	<0.001	1.267
Camel	9 (0.2)	8 (0.2)	1 (0.4)	1	0.022
Raw Milk Consumption–n (%)*					
Any	1,551(36.7)	1,320 (33.4)	231 (86.5)	<0.001	1.273
Cow	1,491 (35.3)	1,267 (32.0)	224 (83.9)	<0.001	1.236
Sheep	1,502 (35.6)	1,275 (32.2)	227 (85.0)	<0.001	1.266
Goat	1,502 (35.6)	1,274 (32.2)	228 (85.4)	<0.001	1.287
Camel	19 (0.4)	16 (0.4)	3 (1.1)	0.237	0.082

* = the following milk types are not mutually exclusive.

** = sheep or goats were conflated into a category described as “shoats” in the questionnaires administered to participants in Busia, but sheep and goats were referred to separately in questionnaires utilized in all other regions. Therefore, all respondents reporting milking behavior with “shoats”, “sheep”, or “goats” were included in an inclusive category of “sheep or goats” for the purpose of this analysis. IQR = interquartile range; 25^th^ and 75^th^ percentile. Percentages may include missing values in each category.

Raw milk exposure factors are reported by behavior (milking versus consumption by ingestion), and by specific animal species. Both milking and consumption behaviors were found to be associated with a significant risk of exposure (any milking behavior by the seropositive group: n = 206 (77.2%), versus that of the seronegative group: n = 1,264 (32.0%) (p < 0.001, SMD = 1.024); any raw milk consumption by the seropositive group: n = 231 (86.5%), versus that of the seronegative group: n = 1,320 (33.4%) (p < 0.001, SMD = 1.273)). In order to identify the varied risks associated with exposures to animal types or milk derived from specific animal species, each behavior was also analyzed by specific animal species. Regardless of village or exposure behavior, cows (n = 1,219 (28.9%)), sheep and goats (n = 1,000 (23.7%)) remained the most commonly raised livestock for milk production. Individuals with a history of RVFV infection (n = 267) reported milking cows (n = 193 (72.3%), p <0.001) and sheep or goats (n = 201 (75.3%), p < 0.001) with similar prevalence. Comparatively, individuals without prior RVFV infection (n = 3,956) reported less milking of cows (n = 1,026 (25.9%)), and sheep or goats (n = 799 (20.2%)), overall. Additionally, consumption of cow’s milk (n = 224 (83.9%), p < 0.001), sheep’s milk (n = 227 (85.0%), p < 0.001), or goat’s milk (n = 228 (85.4%), p < 0.001), was reported with similar regularity by individuals with prior RVFV infection (n = 267). Individuals without prior RVFV infection (n = 4,020) reported consumption of raw milk for approximately one-third of respondents, with a similar reporting frequency for cows (n = 1,267 (32.0%)), sheep (n = 1,275 (32.2%)), and goats (n = 1,274 (32.2%)). Very few respondents reported exposure to raw camel milk through milking (n = 9 (0.2%)) or by consumption (n = 19 (0.4%)), compared to other types of animals reported (Table 1). Milking and consumption odds ratios adjusted for age and gender are presented in S1 Table.

**Fig 2:**
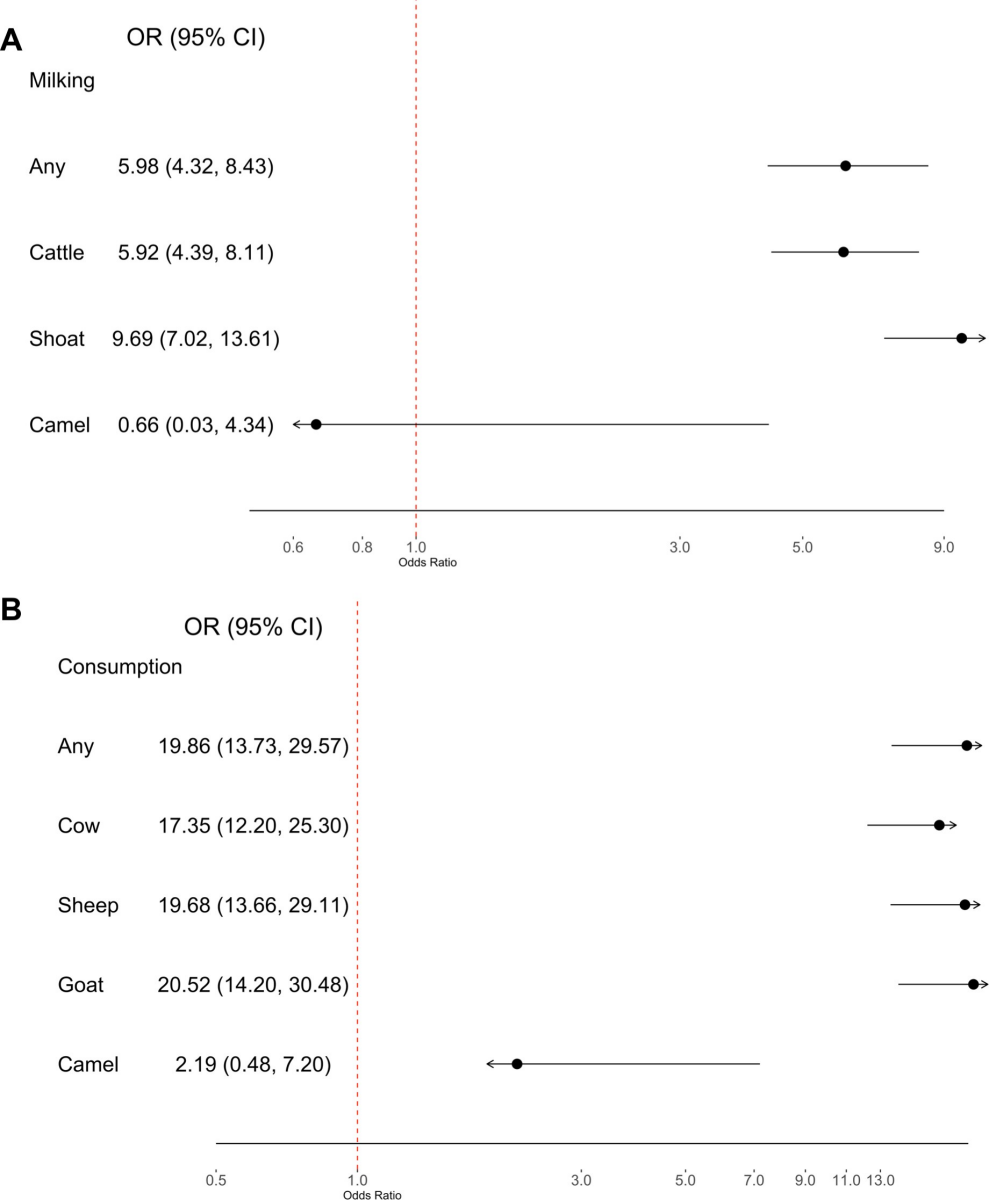
**Forest plot of milking exposure (A) and raw milk consumption (B) by animal type.** Odds ratios reported with CI_95_. Any milking (A) or consumption (B) includes exposure to any or all animal types detailed in the questionnaire. No other animal types were reported. Sheep or goats reported for milking exposures were conflated into a category described as “shoats” in the questionnaires administered to participants in Busia, but sheep and goats were referred to separately in questionnaires utilized in all other regions. Therefore, all respondents reporting milking behavior with “shoats”, “sheep”, or “goats” were confounded in an inclusive category of “sheep or goats” for the purpose of this analysis.

Risk of infection, measured by serological data indicating prior infection, was found to be significant regardless of behavior (Fig 2). No significant difference in exposure risk was found between specific milking behaviors with cows, sheep, or goats (Fig 2A). Exposure to camels by milking was observed to have non-significantly lower odds for RVFV exposure (p = 0.71, OR = 0.66, CI_95_ 0.03–4.34) than that of other animal species (milking cows, p <0.001, OR = 5.92, CI_95_ 4.39–8.11); milking sheep or goats, p <0.001, OR = 9.69, CI_95_ 7.02–13.61) (Fig 2A).

Similarly, risk of exposure by consumption of raw milk was comparable between cow’s milk, sheep’s milk, or goat’s milk (Fig 2B). Consumption of raw camel milk was not found to be a significant transmission risk for RVFV exposure (p = 0.24, OR = 2.19, CI_95_ 0.48–7.20) than those of other animal species (cow’s milk: p <0.001, OR = 17.35, CI_95_ 12.20–25.30; sheep’s milk: p <0.001, OR = 19.68, CI_95_ 13.66–29.11; goat’s milk: p <0.001, OR = 20.52, CI_95_ 14.20–30.48) (Fig 2B). Age- and gender- adjusted odds of RVFV infection by method of exposure and milk type are further described in S1 Table.

In an attempt to identify regionally-specific behaviors, populations included in this analysis were grouped by eastern and western geographical regions within Kenya. Odds ratios were then adjusted by region, in addition to gender and age (Table 2). Variables such as age and gender were included for insight into demographic differences, as well as possible risk factors dependent on geographical region.

**Table 2:** Adjusted odds of RVFV infection including age, gender, and region. Models were adjusted by age, gender, and region. Participants from Busia were included in the “west” cohort, and all other villages were located on the eastern coast of Kenya, thus grouped and designated as the “east” cohort.

Characteristics	aOR (95% CI)	p-value
Milking		
Any		
Age	1.05 (1.04, 1.05)	<0.001
Female (ref = male)	0.94 (0.71, 1.27)	0.70
Region West (ref = East)	0.04 (0.02, 0.06)	<0.001
Milking Duties (ref = no)	1.8 (1.24, 2.64)	0.00
Cattle		
Age	1.05 (1.04, 1.05)	<0.001
Female (ref = male)	0.94 (0.7, 1.26)	0.66
Region West (ref = East)	0.04 (0.02, 0.06)	<0.001
Milking Duties (ref = no)	1.7 (1.2, 2.43)	0.00
Sheep or Goats		
Age	1.05 (1.04, 1.05)	<0.001
Female (ref = male)	0.91 (0.68, 1.23)	0.55
Region West (ref = East)	0.07 (0.03, 0.12)	<0.001
Milking Duties (ref = no)	2.07 (1.4, 3.14)	<0.001
Camel		
Age	1.05 (1.04, 1.06)	<0.001
Female (ref = male)	0.95 (0.71, 1.28)	0.73
Region West (ref = East)	0.04 (0.02, 0.06)	<0.001
Milking Duties (ref = no)	0.24 (0.01, 1.7)	0.22
**Raw Milk Consumption**		
Any		
Age	1.05 (1.04, 1.06)	<0.001
Female (ref = male)	0.9 (0.68, 1.19)	0.45
Region West (ref = East)	0.04 (0.02, 0.1)	<0.001
Consumption (ref = no)	0.99 (0.45, 2.51)	0.99
Cattle		
Age	1.05 (1.04, 1.06)	<0.001
Female (ref = male)	0.9 (0.67, 1.19)	0.44
Region West (ref = East)	0.04 (0.02, 0.09)	<0.001
Consumption (ref = no)	1.02 (0.54, 2.08)	0.95
Sheep		
Age	1.05 (1.04, 1.06)	<0.001
Female (ref = male)	0.89 (0.67, 1.19)	0.43
Region West (ref = East)	0.04 (0.02, 0.1)	<0.001
Consumption (ref = no)	0.96 (0.46, 2.3)	0.92
Goat		
Age	1.05 (1.04, 1.06)	<0.001
Female (ref = male)	0.9 (0.67, 1.19)	0.44
Region West (ref = East)	0.04 (0.02, 0.11)	<0.001
Consumption (ref = no)	1.06 (0.49, 2.7)	0.89
Camel		
Age	1.05 (1.04, 1.06)	<0.001
Female (ref = male)	0.9 (0.67, 1.19)	0.44
Region West (ref = East)	0.04 (0.02, 0.06)	<0.001
Consumption (ref = no)	0.62 (0.13, 2.11)	0.48

In all models, both age and region were distinguished as variables that impacted exposure, regardless of behavior (milking versus consumption or ingestion), or animal type (Table 2). These data indicated a lack of difference in gender roles for milk-related behaviors between western and eastern regions of Kenya. Similarly, child participants (aged between 1 and 15 years of age) were not associated with a higher likelihood of exposure when compared to adult participants (aged 16 to 87 years of age), regardless of behavior.

In order to distinguish the importance of each distinct milk-exposure behavior for RVFV transmission in individuals who engage in both milking and consuming raw milk, each exposure was analyzed for effect relative to the other behavior (Table 3). The top section of Table 3 displays the relative effect of performing milking duties, or lack of milking duties, and how such milking duties affect two behavior groups (consumers and non-consumers), individually. Resulting OR and p-values reported for each row are the odds of being seropositive for RVFV for those that perform milking duties compared to those who do not perform milking duties within each group (consumers or non-consumers). The bottom section of Table 3 displays the relative effect of raw milk consumption for two behavior groups (those who perform milking duties versus those who do not perform milking duties). Milking duties appeared to influence risk in individuals who also consume raw milk (OR 2.3, CI_95_ 1.48–3.59, p < 0.001); however, milking duties do not appear to influence risk for those who did not report consumption of raw milk (OR 0.51, CI_95_ 0.17–1.57, p = 0.24), whereas consumption behaviors did not increase risk of exposure whether the individuals also had milking duties (OR 2.1, CI_95_ 0.59–7.43, p = 0.25) or not (OR 0.47, CI_95_ 0.17–1.27, p = 0.13). Each of these effects was observed in general, animal-nonspecific milking and consuming behaviors, as well as animal-specific exposures in cattle, sheep, and goats, with the exception of camels (Table 3).

**Table 3:** Comparison of individual behavior exposure versus combined behavior exposures. Adjusted by age, gender, and region.

Characteristics	OR (95% CI)	p-value
**Effect of Milking Duties for Raw Milk Consumers/Non-consumers**		
Any		
Consumers	2.31 (1.48, 3.59)	<0.001
Non-consumers	0.51 (0.17, 1.57)	0.24
Cattle		
Consumers	2.06 (1.37, 3.1)	<0.001
Non-consumers	0.83 (0.31, 2.2)	0.71
Sheep		
Consumers	2.28 (1.47, 3.53)	<0.001
Non-consumers	2.24 (0.5, 10.01)	0.29
Goat		
Consumers	2.28 (1.47, 3.54)	<0.001
Non-consumers	2.04 (0.41, 10.08)	0.38
Camel		
Consumers	0.27 (0.02, 4.44)	0.36
Non-consumers	0 (0, Inf)	1
**Effect of Raw Milk Consumption for Milking Duties/No Milking Duties**		
Any		
Milking Duties	2.1 (0.59, 7.43)	0.25
No Milking Duties	0.47 (0.17, 1.27)	0.13
Cattle		
Milking Duties	1.46 (0.56, 3.83)	0.44
No Milking Duties	0.59 (0.26, 1.33)	0.20
Sheep		
Milking Duties	0.66 (0.2, 2.13)	0.49
No Milking Duties	0.65 (0.22, 1.96)	0.44
Goat		
Milking Duties	0.75 (0.2, 2.77)	0.66
No Milking Duties	0.67 (0.22, 2.02)	0.47
Camel		
Milking Duties	60253.72 (0, Inf)	1
No Milking Duties	0.99 (0.21, 4.72)	0.99

## Discussion

Our data illustrate the risk of RVFV transmission associated with raw milk consumption and milking behaviors and reveals the act of milking as a likely significant contributor to viral transmission. The act of milking animals is a culturally, nutritionally, and financially important practice that is performed around the world. Identification of raw milk products and milking behaviors as a potential pathway of RVFV transmission may be critical for ongoing efforts, such as predictive modeling[31, 66], cell and molecular research[6], or clinical and public health interventions[67], to mitigate outbreaks and prevent RVFV emergence into new areas of the world.

Public knowledge of RVFV and its diverse transmission processes is relatively limited, and public health efforts are likely to fall short in encouraging thoroughly safe practices. During outbreaks in eastern Africa in 2018, efforts to limit the spread of RVFV were mainly focused on abattoirs and consumption of meat products. Residents in many affected villages in western Kenya were advised to “only eat inspected livestock products, including milk and meat” [68]. While milk and meat are included in this statement, animal handling activities such as milking were not mentioned. Additionally, potential methods for risk mitigation, such as boiling milk products for sterilization, or mosquito abatement outside of standard mosquito net usage, were not mentioned [68].

Milk is routinely considered as a potential route for zoonotic transmission of RVFV, yet the distinction between risks from milk consumption versus risks from direct animal contact through milking has yet to be defined. Milk is acknowledged as a risk factor for RVFV transmission in many publications [54, 61, 62, 67, 69–75], yet the contributive weight or regularity of milk as a route of transmission [76, 77], and the possibility of milk containing live virus [76, 78] is regularly debated. Consumption of raw or unpasteurized milk is often mentioned as a possible but unusual method of exposure [71, 77, 79]. There is a minimal amount of experimental evidence of live virus actively being shed into the milk of lactating animals [80, 81], but these experiments were limited, and further experimental data from more recent experiments have yet to be published [3]. In this study, we found exposure to raw milk to be correlated with prior RVFV infection. All consumption behaviors were found to be significantly associated with seropositivity after adjustments for age and gender (S1 Table).

Our data describe milking behaviors, regardless of species of animal, as a significant risk factor for exposure to RVFV. Few studies report exposure to raw milk as a potential route for RVFV transmission with a distinction between milking and ingestion or consumption [54, 67, 69, 72]. A study in Madagascar by Olive in 2016 describes the variable of raw milk as “contact with raw milk” in their statistical correlation to serological data [69], which does little to define the true route or behavior influencing increased exposure risk. Occupation is often analyzed as a representation of animal exposure, yet keeping domesticated livestock at the homestead or as a village is common practice throughout sub-Saharan Africa. Occupations with specific types of animal handling, such as slaughterman in an abattoir, have been shown to pose an increased risk of exposure to RVFV through handling of a higher volume of live animals, animal carcasses, and bodily fluids during slaughtering [14]. Additionally, methods associated with slaughtering and carcass cleaning may cause the virus to become aerosolized, leading to an alternative inhalational route of transmission. Of note, exposure via aerosolization has been associated with severe neurological sequelae in mouse models of RVF [82].

Attitudes around and behaviors with milk and types of animals kept is likely to vary among villages, as Kenya is comprised of a vast landscape of ecosystems and human populations with diverse cultures and beliefs. Food and beverages often have very strong cultural and/or ritualistic significance, transcending basic usage for sustenance. As with many foodborne illnesses, processing meats and animal byproducts, whether by thoroughly cooking, preserving, or other sterilization methods, reduces the likelihood of transmission of RVFV. For example, processing of meat products results in a change in pH in the tissues and fluids which effectively inactivates live virus [46, 83]. Simulated experiments have shown select viruses can be propagated in milk and milk products, such as heavy cream and ice creams [84]. Many cultures use foods directly as or for the administration of therapeutics. In a study by Mutua *et al*., consumption of a variety of animal products was common, yet meat and milk specifically were used in the administration of medicines [67], which adds to the complexity of behaviors and potential routes for RVFV transmission. Consideration of the reason for specific animal fluids or tissues ingestion, and whether the products have been processed, cooked, or are being ingested raw should be included in future foodborne RVFV transmission research.

When analyzed by species, cattle and sheep or goats, referred to as “shoats” in this analysis, were found to be a significantly associated with prior infection, as indicated by seropositivity. Behaviors relating to exposure to camel milk were not found to be significantly associated with seropositivity in our analysis. These findings may allude to chemical or molecular differences in milks produced by different animal species that could potentially influence the viability of RVFV in solution. Further research is necessary to properly characterize potential differences between milks from different species. It is also important to note that milk may be a risk factor, not only for humans, but also for nursing animals. Perhaps the high susceptibility of young ruminants for severe and deadly RVF is due to double RVFV exposure in both vector and mother’s milk. While horizontal transmission in animals and humans has yet to be proven, more research is needed to investigate likelihood of non-vector-borne transmission in animals, such as the possible competence of milk to allow horizontal transmission in ruminant species.

It is unclear whether men or women are at higher risk for exposure. Our data did not uncover a direct correlation between gender and milking or milk consumption behaviors, yet others have reported behaviors significantly linked to gender. Mutua *et al*. describe that while milking is primarily a responsibility of women, women were less likely to engage in milking with sick animals. Conversely, men were more likely to consume milk from sick animals. This suggests that the differences reported in many studies regarding one gender as a risk factor over the other may be referring to gender roles and gender differences by behavior dictated by differing cultures.

This study has number of limitations that should be considered in future studies. The questionnaire responses analyzed were comprised of self-reported data regarding personal behaviors of the participants and may be subject to recall bias. This study only includes data from two geographically opposite regions of Kenya, and other endemic or potentially-exposed regions may experience differential transmission based on the animal species that are most commonly kept for milking or from which animal milk is sourced for consumption. Access to species-specific milks or products may vary in other regions that are endemic for RVFV, or at risk for future RVFV emergence. In addition to species-specific milk access, pasteurized or boiled milk was not discussed in this study, and questionnaires utilized in each study only asked for behaviors around raw milk exposure. Source of milk consumed was not collected in these questionnaires, and exposure risks through milking for one’s own consumption in their homestead or village may be different than that of people who buy raw milk for consumption. Additional considerations will be dedicated to the trade and sale of raw milk in relation to transmission of RVFV. Lastly, only a small number of participants from our study reported exposures to camels with relation to milk behaviors, which may not accurately represent exposure to camel milk throughout Kenya, or in other RVFV endemic regions.

There are many public health strategies for outbreak mitigation that may be improved with the inclusion of raw milk products and milking behaviors as potential risk factors for RVFV transmission. Epidemiological investigations relating to the origin and spread of an outbreak should include questions about milk consumption and milking behaviors. Survey questions relating to milk consumption should relate to specifically raw milk, as well as personal sterilization or pasteurization efforts, such as boiling or fermentation, to ensure proper techniques are being used. Bulletins distributed during outbreaks should include raw milk as a potential risk, and should encourage individuals to avoid milking animals that are showing symptoms of infection or that have been exposed to other animals with suspected or confirmed infection[64, 67]. Current bulletin efforts readily list the consumption of blood, tissues, and raw meat as a route of exposure, yet could improve awareness around other animal-related exposures[11, 64]. As further research is conducted, public education and community awareness efforts administered by local clinics and ministries of health should be updated to include a more extensive list of risks[67]. Specific attention should continue to be directed towards livestock trade as potential routes for viral spread into new communities and territories[25, 26, 76, 85–87]. It may be worthwhile to instate policies and guidelines for economies relating to the sale and shipment of raw milk products at the local level, as seen with current guidelines relating to international and inter-country livestock trade and animal product sales[49, 88].

This study illustrates the potentially significant influence of exposure to raw milk on RVFV exposure, and the importance of direct animal contact in non-vector-borne transmission cycles. Milk exposures are often involved in everyday behaviors, such as occupation, cultural or ritualistic practices, and therefore increase the complexity of transmission risks and the level of detail required for implementing effective risk mitigation. A heightened understanding of the differential risks associated with meats and milk products from various species is necessary to fully describe the transmission cycle for RVFV. The inclusion of raw milk exposure and milking tasks as risk factors for transmission may explain the high level of variability in incidence and prevalence among villages. Further efforts should be dedicated to viral isolation studies for characterization of the role of milk in interepidemic transmission and maintenance of the RVFV. Milk risks may also impact the likelihood of emergence into naïve populations, with risk of importation via trade without regulation of milk products.

## Supporting information

S1 Table Age- and gender-adjusted odds of RVFV infection by method of exposure and milk type. * = sheep or goats were conflated into a category described as “shoats” in the questionnaires administered to participants in Busia, but sheep and goats were referred to separately in questionnaires utilized in all other regions. Therefore, all respondents reporting milking behavior with “shoats”, “sheep”, or “goats” were included in an inclusive category of “sheep or goats” for the purpose of this analysis.
(DOCX)
