# Supplementary material for: The influence of raw milk exposures on Rift Valley fever virus transmission
Source: PLoS Negl Trop Dis. 2019 Mar 20;13(3):e0007258. doi: 10.1371/journal.pntd.0007258 (PMC6443189; doi:10.1371/journal.pntd.0007258)
Supplement: S1 Table — * = sheep or goats were conflated into a category described as “shoats” in the questionnaires administered to participants in Busia, but sheep and goats were referred to separately in questionnaires utilized in all other regions. Therefore, all respondents reporting milking behavior with “shoats”, “sheep”, or “goats” were included in an inclusive category of “sheep or goats” for the purpose of this analysis. (DOCX) [file pntd.0007258.s001.docx]

**S1 Table.** **Age- and gender-adjusted odds of RVFV infection by method of exposure and milk type**. * = sheep or goats were conflated into a category described as “shoats” in the questionnaires administered to participants in Busia, but sheep and goats were referred to separately in questionnaires utilized in all other regions. Therefore, all respondents reporting milking behavior with “shoats”, “sheep”, or “goats” were included in an inclusive category of “sheep or goats” for the purpose of this analysis.

| **Characteristics** | **OR (CI_95_)** | ***p* value** |
| --- | --- | --- |
| Milking – n (%) |  |  |
| Any | 5.98 (4.32, 8.43) | <0.001 |
| Cow | 5.92 (4.39, 8.11) | <0.001 |
| Sheep or Goat* | 9.69 (7.02, 13.61) | <0.001 |
| Camel | 0.66 (0.03, 4.34) | 0.71 |
|  |  |  |
| Raw Milk Consumption – n (%) |  |  |
| Any | 19.86 (13.73, 29.57) | <0.001 |
| Cow | 17.35 (12.20, 25.30) | <0.001 |
| Sheep | 19.68 (13.66, 29.11) | <0.001 |
| Goat | 20.52 (14.20, 30.48) | <0.001 |
| Camel | 2.19 (0.48, 7.20) | 0.24 |
|  |  |  |
